# Effects of sildenafil, metformin, and simvastatin on ADH‐independent urine concentration in healthy volunteers

**DOI:** 10.14814/phy2.13665

**Published:** 2018-04-02

**Authors:** Anneke P. Bech, Jack F. M. Wetzels, Tom Nijenhuis

**Affiliations:** ^1^ Department of Nephrology Radboud University Medical Center Nijmegen The Netherlands

**Keywords:** Metformin, nephrogenic diabetes insipidus, sildenafil, simvastatin, urine concentration

## Abstract

Nephrogenic diabetes insipidus (NDI) is a rare disorder characterized by resistance of the kidney to the action of antidiuretic hormone (ADH), resulting in a decrease in the capacity of the kidney to concentrate the urine. NDI can be inherited or acquired due to, for example, chronic lithium therapy. Current treatment options are limited to attempts to lower urine output by a low‐solute diet and the use of diuretics or anti‐inflammatory drugs. These measures are only partially effective. Recent reports suggested that sildenafil, metformin, and simvastatin might improve ADH‐independent urine concentration. If confirmed, this would provide interesting additional therapeutic options for patients with NDI. We, therefore, tested the effect of these drugs on ADH‐independent urine concentrating capacity in healthy volunteers. We included 36 healthy volunteers who received sildenafil 20 mg thrice daily, metformin 500 mg thrice daily or simvastatin 40 mg once daily during 1 week. At baseline and at the end of treatment, a water loading test was performed. No significant increase in lowest urine osmolality was seen after the use of metformin or sildenafil (*P* = 0.66 and *P* = 0.09 respectively). Lowest urine osmolality increased modestly but significantly after the use of simvastatin (70 mOsm/kg to 85 mOsm/kg, *P* = 0.05). Our data suggest that only simvastatin has an effect on urine osmolality in healthy volunteers. Validation studies are needed and, most importantly, these drugs should be tested in patients with NDI.

## Introduction

**Figure 1:**
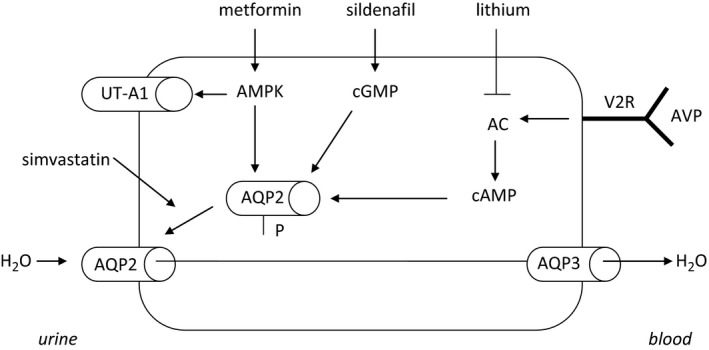
Overview of potential therapeutic targeting of the intracellular mechanisms involved in transcellular water reabsorption in the collecting duct. Upon vasopressin (AVP) binding to its receptor (V2R) at the basolateral side of the principal collecting duct cells, an intracellular signaling cascade is activated that ultimately results in phosphorylation and subsequent insertion of aquaporin‐2 water channels (AQP2) in the apical membrane. Sildenafil is hypothesized to target this cascade by increasing cyclic guanosine monophosphate (cGMP), metformin by increasing adenosine monophosphate‐activated protein kinase (AMPK) and simvastatin through inhibiting the recycling of AQP2 and thereby a net increase in AQP2 channels inserted in the membrane. UT‐A1, urea transporter A1; AC, adenylyl cyclase; V2R, vasopressin receptor type 2; AVP, arginine vasopressin.

Nephrogenic diabetes insipidus (NDI) is a rare disorder characterized by resistance of the kidney collecting duct to the action of antidiuretic hormone (ADH), resulting in a decrease in the capacity of the kidney to concentrate urine. Water reabsorption in the collecting duct is initiated by ADH that binds to its receptor (type 2 vasopressin receptor, V2R) in the collecting duct principal cells (Fig. 1). Activation of the V2R activates an intracellular signaling cascade in which stimulation of adenylyl cyclase (AC) leads to the production of cyclic adenosine monophosphate (cAMP), which in turn stimulates the insertion of aquaporin‐2 water channels (AQP2) into the apical membrane through which water can enter the cell. Hereditary NDI is caused by mutations in the *AVPR2* gene (encoding for the V2R) in the vast majority of patients, leading to X‐linked NDI. Alternatively, mutations in the *AQP2* gene (encoding aquaporin‐2) can cause either autosomal‐dominant or recessive NDI, or it can occur in the context of hereditary tubulointerstitial kidney diseases. The most common cause of acquired NDI is chronic lithium therapy. The inability to concentrate urine results in high urine volumes, which can be as high as 15 L per day in patients with hereditary NDI. This can lead to several secondary problems, such as hypernatremia and rapid dehydration when water intake is restricted. In addition, chronic vesicoureteral reflux leading to chronic kidney injury can occur due to chronically high bladder volumes. Current treatment consists of countering the polyuria by drinking large amounts of water and attempts to lower urine output by a low‐salt and low‐protein diet, the use of nonsteroidal anti‐inflammatory drugs and the use of diuretics that increase proximal sodium and water reabsorption and/or prevent lithium influx into principal cells (Crawford et al. 1960; Forrest et al. 1974; Kortenoeven et al. 2009; Bockenhauer and Bichet 2015). These measures, however, are only partly able to correct the polyuria. Therefore, NDI is a chronic disease seriously affecting the quality of life.

Recent reports have suggested novel therapeutic options by repurposing already available and FDA approved drugs that could, theoretically, be more effective than the current standard of care (Sands and Klein 2016). The rationale for using these drugs in the context of NDI is based on the fact that they would be able to beneficially target the intracellular signaling cascade downstream to the V2R receptor in the collecting tubule cell and thereby potentially rescue, at least in part, urine concentrating ability (Fig. 1). The first drug for which a potential positive effect on urine concentration was suggested is sildenafil, currently registered for use in erectile dysfunction and pulmonary hypertension. Sildenafil increases cyclic guanosine monophosphate (cGMP) which, like cAMP, is thought to be able to induce phosphorylation of AQP2 and thereby promote insertion of AQP2 water channels into the apical membrane (Fig. 1). Sildenafil indeed increased the apical accumulation of AQP2 in rats with central diabetes insipidus (Bouley et al. 2005; Sanches et al. 2012). Assadi and Sharbaf (2015) described a child with a mutation in V2R who showed a lower urine volume and higher urine osmolality after 10 days treatment with sildenafil compared to conventional treatment with hydrochlorothiazide, amiloride, and indomethacin. The second potential drug is metformin, currently registered for use in diabetes mellitus. Metformin activates adenosine monophosphate kinase (AMPK), which increases phosphorylation and accumulation of AQP2 (Fig. 1). Metformin resulted in an increase in urine osmolality in V2R knockout mice and tolvaptan‐treated rats (Efe et al. 2016; Klein et al. 2016). The third potential drug is simvastatin, a cholesterol‐lowering drug of the HMG‐CoA‐reductase inhibitor class of drugs. With respect to NDI, simvastatin is thought to enhance the expression of AQP2 through down regulation of Rho GTPases and the inhibition of AQP2 endocytosis (Fig. 1) (Li et al. 2011). In line with this finding, it was shown that hypercholesterolemic patients upon initiation of simvastatin show an increase in urinary AQP2 and an increase in urine osmolality (Procino et al. 2016).

These three drugs are thus hypothesized to have an ADH‐independent effect on AQP2 and water transport in the collecting duct, where statins are mainly thought to promote AQP2 trafficking to the membrane and sildenafil and metformin would induce phosphorylation of AQP2. These drugs could thereby serve as new treatment options in patients with NDI, particularly by bypassing the defective V2R receptor signaling in patients with *AVPR2* mutations. In this study, we performed physiological experiments determining whether these drugs affect ADH‐independent urine concentration in healthy man, which would strengthen their therapeutic potential in patients with NDI.

## Methods

### Study population

We performed a study in 36 healthy volunteers, aged >18 year and not using any medication. After informed consent, each healthy volunteer received either sildenafil (20 mg thrice daily), metformin (500 mg thrice daily) or simvastatin (40 mg once daily) during 1 week. In total, 12 healthy volunteers per study medication were included. A water loading test was performed to evaluate ADH‐independent urine concentration/dilution. This study had a cross‐over design, that is, each patient was studied after 1 week on and off therapy with at random selection of the order of the study periods.

The tests were performed in the Radboud university medical center by a group of trained nurses. This study was approved by the medical ethics committee of the Radboud university medical center Nijmegen, and all participants provided written informed consent.

### Water loading test

In the 24 h preceding the test, the subjects were not allowed to smoke cigarettes or drink alcohol or coffee. On the morning of the test, subjects were allowed to have a small breakfast without coffee or tea. They were instructed to drink two glasses of water before going to bed and to drink an extra glass of water at breakfast.

The healthy volunteers then visited the clinic at 8:00 h in the morning. At that time (*T* = 0), subjects were weighed, blood pressure was measured and a urine sample was taken. Next they were requested to lie down for one hour. At *T* = 60 (min), a urine and blood sample were taken and the subject was instructed to drink 20 mL/kg of body weight in 15 min. Thereafter, every hour, urine samples were collected during 4 h. At *T* = 300, an additional blood sample was withdrawn and body weight and blood pressure were measured again. During the test, participants were not allowed to eat, to drink coffee or tea, and they were not allowed to smoke cigarettes. Except for going to the toilet, the subjects were seated and not allowed to walk around.

### Statistics

Baseline characteristics are reported as median values with interquartile ranges. Medians between the baseline test and the test after medication were compared, using a Wilcoxon matched pairs test. Spearman correlation coefficients were used to perform univariate analyses. Statistical significance was defined as a two‐sided *P* ‐value of <0.05. Statistical analyses were performed, using SPSS.

### Power calculation

We considered an increase in lowest osmolality of more than 20 mOsm/kg clinically relevant. A power calculation with a two‐sided paired *T* test with a power of 0.80, an alpha of 0.05 results in a number of 12 subjects per drug.

## Results

**Figure 2:**
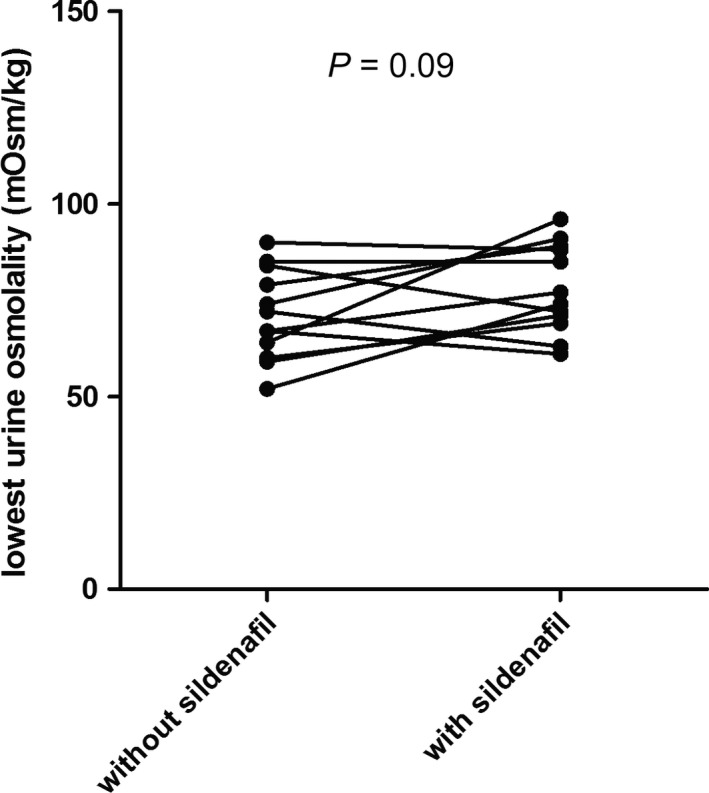
Effect of sildenafil on urine osmolality after a water loading test in healthy subjects. The lowest urine osmolality after water loading in healthy subjects is depicted either with or without prior treatment with sildenafil (20 mg thrice daily) during 1 week.

Test results of the subjects on sildenafil are shown in Table 1 and Figure 2. Eight out of twelve subjects were male. Median age was 27 years (IQR 22–34). Urine osmolality at start of the water loading test was lower on the test day after sildenafil compared to the test day without sildenafil, but this difference was not significant. Lowest urine osmolality was not significantly higher after the use of sildenafil (+6 mOsm/kg, +9%, *P* = 0.09). Frequent side effects were noted during the treatment period. Six subjects experienced headache, four subjects blushing, one subject diarrhea, one subject palpitations, one subject dizziness, and four subjects did not experience side effects.

**Table 1:** Test characteristics in the sildenafil group

	Without sildenafil	With sildenafil	*P* value
Body weight (kg) – start	73 (63–90)	73 (64–89)	0.18
Body weight (kg) – end	72 (63–90)	72 (64–92)	0.05
Systolic BP – start	123 (120–134)	122 (112–132)	0.05
Systolic BP – end	124 (115–129)	128 (111–132)	0.42
Diastolic BP – start	73 (64–77)	66 (61–73)	0.37
Diastolic BP – end	70 (66–80)	72 (59–76)	0.19
Pulse – start	75 (61–91)	82 (70–91)	0.21
Pulse – end	61 (53–66)	62 (57–70)	0.25
Serum creatinine – start (μmol/L)	79 (63–89)	73 (68–88)	0.17
Urine osmol baseline (mOsm/kg)	441 (262–618)	226 (105–540)	0.18
Lowest urine osmol (mOsm/kg)	70 (61–83)	76 (70–89)	0.09
Sodium excretion (mmol in 300 min)	44 (36–52)	55 (50–64)	0.06
Potassium excretion (mmol in 300 min)	19 (15–25)	25 (21–32)	0.02
Urea excretion (mmol in 300 min)	95 (74–125)	100 (90–125)	0.43

Median values with IQR.

**Figure 3:**
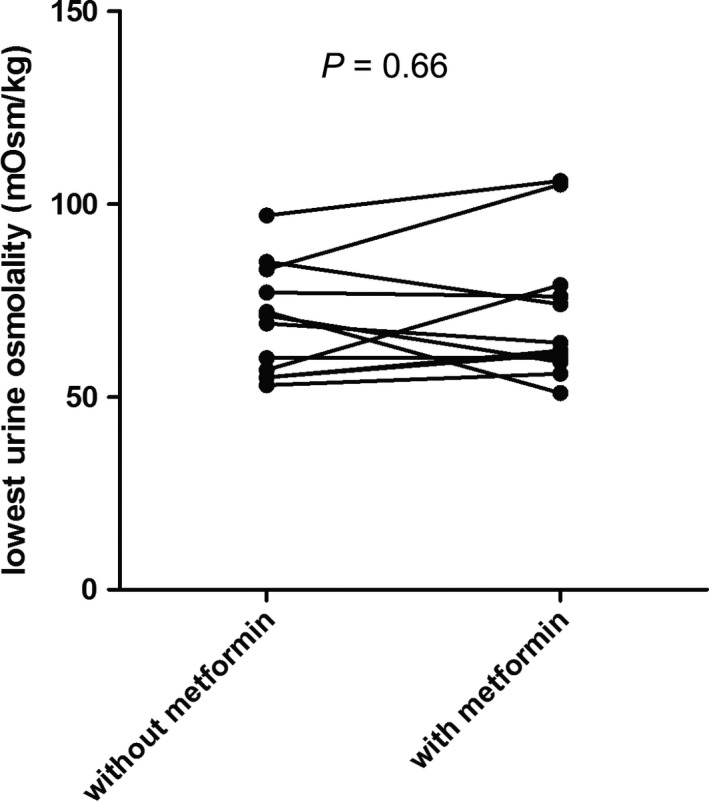
Effect of metformin on urine osmolality after a water loading test in healthy subjects. The lowest urine osmolality after water loading in healthy subjects is depicted either with or without prior treatment with metformin (500 mg thrice daily) during 1 week.

Test results of the subjects on metformin are shown in Table 2 and Figure 3. Five out of twelve subjects were male. Median age was 20 years (IQR 19–25). Urine osmolality at start of the water loading test was lower on the test day after metformin compared to the test day without metformin, but this difference was not significant. Lowest urine osmolality was not different after the use of metformin (−7 mOsm/kg, −10%, *P* = 0.66). Two subjects did not experience any side effect, five subjects had diarrhea, and eight subjects experienced nausea when using metformin.

**Table 2:** Test characteristics in the metformin group

	Without metformin	With metformin	*P* value
Body weight (kg) – start	69 (58–78)	69 (59–77)	0.27
Body weight (kg) – end	68 (58–77)	68 (58–77)	0.08
Systolic BP – start	117 (111–128)	115 (110–124)	0.39
Systolic BP – end	126 (111–140)	116 (107–128)	0.12
Diastolic BP – start	66 (60–72)	67 (61–70)	0.61
Diastolic BP – end	63 (58–74)	70 (60–72)	0.92
Pulse – start	77 (69–89)	71 (61–79)	0.01
Pulse – end	63 (60–72)	61 (59–66)	0.24
Serum creatinine – start (μmol/L)	73 (62–78)	73 (68–81)	0.13
Urine osmol baseline (mOsm/kg)	368 (153–651)	188 (87–651)	0.75
Lowest urine osmol (mOsm/kg)	70 (56–82)	63 (59–78)	0.66
Sodium excretion (mmol in 300 min)	43 (36–49)	35 (31–49)	0.21
Potassium excretion (mmol in 300 min)	23 (17–27)	17 (16–23)	0.24
Urea excretion (mmol in 300 min)	91 (79–102)	75 (66–97)	0.12

Median values with IQR.

**Figure 4:**
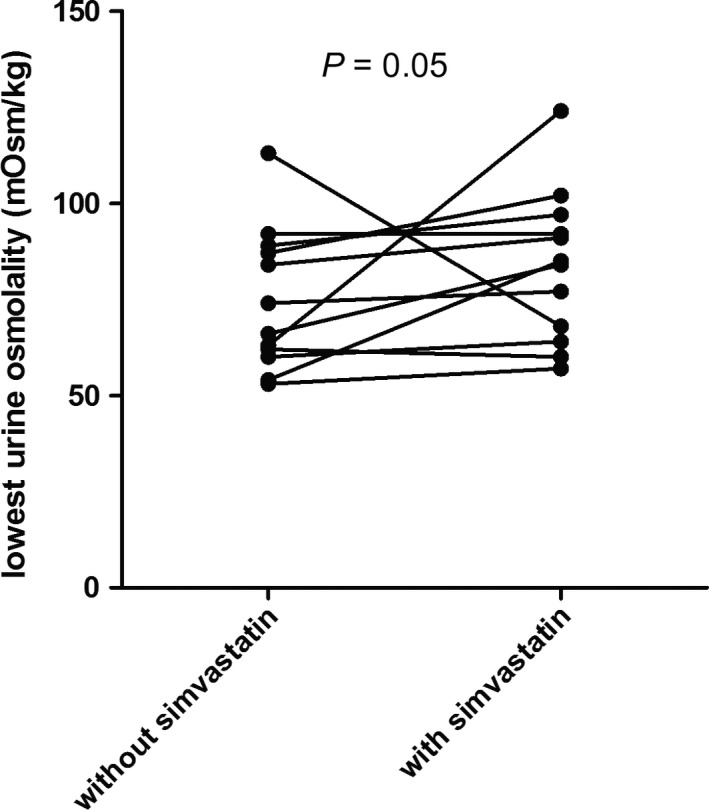
Effect of simvastatin on urine osmolality after a water loading test in healthy subjects. The lowest urine osmolality after water loading in healthy subjects is depicted either with or without prior treatment with simvastatin (40 mg once daily) during 1 week.

Test results of the subjects on simvastatin are shown in Table 3 and Figure 4. Three out of twelve subjects were male. Median age was 23 years (IQR 20–26). Urine osmolality at start of the water loading test was significantly lower on the test day after simvastatin compared to the test day without simvastatin. Despite this, lowest urine osmolality was significantly higher after the use of simvastatin (+15 mOsm/kg, +21%, *P* = 0.05). One subject experienced abdominal pain and one subject experienced headache during treatment with simvastatin.

**Table 3:** Test characteristics in the simvastatin group

	Without simvastatin	With simvastatin	*P* value
Body weight (kg) – start	67 (59–75)	68 (59–74)	0.94
Body weight (kg) – end	67 (58–76)	67 (58–73)	0.50
Systolic BP – start	122 (113–132)	121 (114–133)	0.62
Systolic BP – end	118 (111–124)	120 (115–124)	0.70
Diastolic BP – start	70 (66–74)	71 (65–79)	0.97
Diastolic BP – end	66 (61–74)	69 (61–76)	0.81
Pulse – start	78 (75–90)	81 (62–90)	0.27
Pulse – end	61 (55–77)	61 (53–77)	0.67
Serum creatinine – start (μmol/L)	79 (68–92)	71 (61–87)	0.02
Urine osmol baseline (mOsm/kg)	760 (353–937)	388 (187–388)	0.02
Lowest urine osmol (mOsm/kg)	70 (61–89)	85 (65–96)	0.05
Sodium excretion (mmol in 300 min)	44 (36–51)	54 (43–61)	0.11
Potassium excretion (mmol in 300 min)	20 (12–32)	26 (13–35)	0.45
Urea excretion (mmol in 300 min)	90 (74–100)	101 (77–110)	0.33

Median values with IQR.

**Figure 5:**
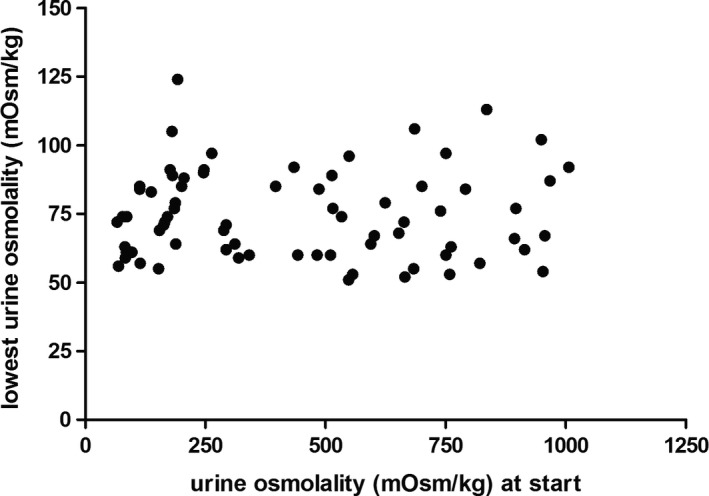
Correlation of baseline urine osmolality and lowest urine osmolality during the water loading test. Depicted is the lowest urine osmolality measured during the water loading test as a function of the urine osmolality measured at the start of the water loading test. Spearman's rho = 0.02, *P* = 0.86.

As the urine osmolality at start of the water loading tests differed between “baseline” and after the week on study drugs in a significant number of subjects, we further studied the relation between the urine osmolality at start and the lowest urine osmolality. Overall, no correlation was seen between urine osmolality at start and at the end of the water loading tests (Fig. 5, Spearman's rho = 0.02, *P* = 0.86).

## Discussion

In this study, we tested whether sildenafil, simvastatin or metformin stimulated ADH‐independent urine concentration in healthy volunteers. Based on the pathophysiological mechanisms and studies in animal models it was expected that these drugs could increase ADH‐independent water reabsorption.

We observed a significant, although limited, increase in lowest urine osmolality after simvastatin. No significant differences were observed with sildenafil or metformin. To appreciate the clinical relevance of the 21% higher urine osmolality with simvastatin, the effects should be compared with that of standard diuretic therapy. Hydrochlorothiazide reduces water requirement by 24–47% (Crawford et al. 1960; Forrest et al. 1974). Although relatively limited in extent, the use of simvastatin could be beneficial to patients with NDI by reducing urine production by approximately 20%. Obviously, our data need to be confirmed and validated in patients with NDI.

The lack of an effect of sildenafil and metformin seems in contrast to earlier reports. Of note, the effects of the three drugs have been mainly tested and demonstrated in animal models.

Effects of metformin on urine concentration were only studied in rodent models. Treatment with 800 mg/kg/day metformin for 4 days increased collecting duct expression of AQP2, increased urine osmolality by 80% in tolvaptan‐treated rats and increased urinary osmolality by 31% in healthy rats (although this increase was not significant with a *P* ‐value of 0.08) (Efe et al. 2016). Already a single dose of 600 mg/kg metformin increased urine osmolality by 180–200% in V2R knock‐out mice (Efe et al. 2016; Klein et al. 2016).

A 3‐week treatment with sildenafil did not affect urine osmolality in healthy rats (Sanches et al. 2012). In contrast, a single dose of 4 mg/kg sildenafil increased kidney tubular AQP2 expression in Brattleboro rats, a model of central diabetes insipidus (Bouley et al. 2005). In support of its efficacy, 200 mg sildenafil per kg food administered for 3 weeks to rats with lithium‐induced NDI, increased urine osmolality by 37% (Sanches et al. 2012).

Brattleboro rats were also used to evaluate the effects of simvastatin on kidney water handling. A single dose of simvastatin increased urine osmolality by ~70% (Li et al. 2011). A single injection of fluvastatin resulted in a modest decrease in urine production in V2R‐mutant mice (Procino et al. 2014). If these same mice were pretreated with secretin, which activates AQP2 gene transcription and thereby increases intracellular AQP2 stores, fluvastatin resulted in a ~90% reduction in urine output (Procino et al. 2014).

Overall, the number of studies in animal models is limited, and only one study evaluated the effect of prolonged treatment. Another important observation is that the vast majority of studies were performed in animal models for diabetes insipidus, whereas the studies in healthy animals did not show a significant effect. Differences in drug dosing could also be relevant. Although we cannot exclude that higher dosages of the drugs could be more effective, for metformin and sildenafil the use of a higher dose does not seem feasible in view of the many known side effects, as also reported by our volunteers. Obviously, it is difficult to compare these studies in animal models with our water loading test in healthy humans.

The potential role of sildenafil and simvastatin in the treatment of NDI was supported by few studies in humans and, thus, the evidence base is very limited. Sildenafil in a dose of 2 mg/kg/day for 10 days was used in a child with NDI due to a mutation in the V2R (Assadi and Sharbaf 2015). Conventional therapy with hydrochlorothiazide, amiloride and indomethacin were continued. Treatment with sildenafil was associated with an increased urine osmolality (from 104 to 215 mosmol/kg) and a parallel reduction in urine output (from 1764 to 950 mL). Data on prolonged treatment and follow up are lacking. There is evidence that simvastatin could positively affect urine concentration capacity in humans. Procino et al. (2016) started simvastatin 20 mg/day in patients with hypercholesterolemia. Within 1 week, urine AQP2 and urine osmolality increased by 36%, an effect that was maintained for 12 weeks. Urine production concomitantly decreased, although to a much lesser degree. Although the authors suggest that their data provide evidence that simvastatin has pleiotropic effects and influences water reabsorption, the data can be interpreted differently. No information is given on food and water intake, both important parameters of urine output. Reduced water intake will simultaneously increase urine osmolality and urine AQP2 excretion. The authors do not provide serum sodium levels, which would allow differentiating between ADH‐dependent or independent increased water reabsorption. Moreover, as a control group, the authors included patients who had been treated with simvastatin for 1 year. Diuresis in these patients was not different than diuresis of treatment‐naive patients.

Despite the overall lack of an effect with metformin and sildenafil in our study, we suggest that testing a combination of drugs such as simvastatin and sildenafil could be considered. As statins mainly promote AQP2 trafficking to the membrane and sildenafil or metformin induce phosphorylation of AQP2, using a combination of both a statin with sildenafil or metformin could theoretically have a synergistic effect. Procino supports this hypothesis by showing that fluvastatin alone does not result in a clinically significant decrease in urine output in V2R‐mutant mice but when it was combined with secretin, which increases intracellular stores of AQP2, it induced a significant decrease in urine output (Procino et al. 2014).

The most important limitations of our study are the trial design which is open, not blinded, and the inclusion of healthy subjects as opposed to NDI patients. The lack of an effect in healthy volunteers does not rule out a potential effect in patients with NDI as acute water loading in healthy subject can possibly result in different ADH‐related changes than in patients with NDI who have a chronically down‐regulated ADH system. Another limitation is that we did not control dietary intake of our subjects. Large differences in osmolar intake can affect minimal urine osmolality (Kleeman et al. 1956). Although we observed slight differences in sodium and urea excretion between the two tests within individual subjects, these differences were not statistically significant. More importantly, if we take these differences into account, the effects of sildenafil and simvastatin on urine osmolality would be even less evident. This supports our conclusion that the drugs are not effective in affecting urine osmolality in healthy subjects.

In conclusion, although insight in the pathophysiological mechanisms provides arguments that metformin, sildenafil, and simvastatin could increase ADH‐independent water reabsorption, experimental evidence is very limited. Our data suggest that simvastatin might have an effect in healthy volunteers. Validation studies are needed and, most importantly, these drugs should be tested alone and in combination in patients with NDI.

## Conflict of Interest

None declared.
